# What Goes In Must Come Out: A Mixed-Method Study of Access to Contraceptive Implant Removal Services in Ghana

**DOI:** 10.9745/GHSP-D-20-00013

**Published:** 2020-06-30

**Authors:** Rebecca Callahan, Elena Lebetkin, Claire Brennan, Emmanuel Kuffour, Angela Boateng, Samuel Tagoe, Anne Coolen, Mario Chen, Patrick Aboagye, Aurélie Brunie

**Affiliations:** a FHI 360, Durham, NC, USA.; b RTI International, Research Triangle Park, NC, USA.; c Population Council, Ghana.; d Ghana Health Service, Family Health Division, Accra, Ghana.; e Marie Stopes International Ghana, Accra, Ghana.; f FHI 360, Washington, DC, USA.

## Abstract

**Background:**

Access to quality removal services is a key component of informed choice in contraceptive implant use; however, limited data exist on users’ access to removal services. In Ghana, implants are available across the country and are the most commonly used contraceptive method among married women.

**Methods:**

From October 2017 and January 2018, we conducted a phone survey with a stratified random sample of 1,159 women who had obtained an implant from a public-sector Ghana Health Service clinic in 2 regions and 1,073 women who had an implant inserted through Marie Stopes International Ghana (MSIG) mobile outreach in 2 other regions. We also interviewed 50 women just after receiving an implant removal from MSIG. We conducted follow-up in-depth interviews with 20 implant acceptors and 15 implant providers across the 4 study regions.

**Results:**

More than four-fifths of women in both service delivery contexts knew that their implant could be removed before its labeled duration. Nearly half of public sector clients and one-third of outreach clients reported that their provider only told them of removal access at the place of insertion. Among women obtaining their implant in the public and outreach sectors, respectively, 32% and 21% reported ever wanting it removed and 61% and 55% who attempted removal obtained a removal on the first attempt. An additional 17% in each context were successful in having their implant removed within 1 week of the first attempt. Most women obtained removal from the same place they received their insertion (81% public, 70% outreach). Most women reported their overall removal experience was very or somewhat easy (74% public, 68% outreach). Challenges included cost, provider availability, interactions with providers, and difficult removals.

**Conclusions:**

Access to implant removal is not universal in Ghana. Strengthening removal services in both the public and outreach sectors is needed to ensure comprehensive access.

## Abstract (precis)

Many Ghanaian women seeking implant removal are able to obtain services, but knowledge and access gaps exist.

## Teaser

**Key Findings:**

- Although most contraceptive implant users in Ghana are able to access removal services, barriers, including cost, provider reluctance to remove, and difficult removals, exist.
- Providers express confidence in removing implants, but many are less confident in their ability to perform difficult removals.

**Key Implications:**

- Family planning programs should review and strengthen potential gaps in removal service provision including access to removals for outreach clients, financial disincentives for removal, and provider training and referral systems for difficult removals.
- More research is needed to assess the prevalence and consequences of difficult implant removals in different settings.

## INTRODUCTION/BACKGROUND

Contraceptive implants are increasingly becoming an integral part of the contraceptive method mix worldwide. Many countries in sub-Saharan Africa, in particular, are rapidly scaling up implant provision. Since the implementation of the Implant Access Program in 2013, which reduced the price of these implants by half, 53 million implants have been purchased for low-resource countries.^1^ In several countries, including Burkina Faso, the Democratic Republic of the Congo, Ethiopia, and Ghana, implants now make up one-quarter to one-half of all modern method use.^2^

Millions of implants are being inserted, but the extent to which providers and programs are prepared to handle the inevitable increase in demand for implant removal services is less clear. Ensuring access to quality implant removal services at term (labeled duration) or at any other time of a woman’s choosing is key for the long-term success of contraceptive implant programs and, even more importantly, compliance with principles of voluntarism and informed choice in contraceptive adoption and use.^3^ Past experience with Norplant in the United States, as well as in low-income countries, demonstrated that lack of access to implant removal services or even the perception of insufficient access can be detrimental to the reputation of the method and raise serious concerns about freedom in contraceptive use.^4^ Anecdotal information from a variety of contexts, as well as some survey data,^5^^–^^7^ points to potential weaknesses in service delivery programs related to implant removal including inadequate medical equipment, insufficient numbers of trained providers, excessive fees required for removal or provider bias against removal before the product’s labeled duration of use. At present, however, systematic data on the accuracy or prevalence of these potential barriers are lacking.

At the global level, recognition of the need for focused attention on implant removal resulted in the creation of the Implant Removal Task Force in 2015.^3^ Although the task force has been instrumental for identifying research and programming gaps, a dearth of data on the state of removal services, especially from users’ perspectives, remains.

We designed this study to generate evidence on the state of access to removal services for women receiving implants in Ghana and to identify areas for improvement that could inform programs globally. We chose Ghana for this study because it is one of the top implant procuring countries in the world^8^ and had unique programmatic data in the form of electronic medical records, which allowed for identification and sampling of implant acceptors. To provide a comprehensive picture of the current situation in Ghana, we examined 2 important service delivery contexts for implants: public sector provision through static facilities and mobile outreach services. Both the Ghana Health Service (GHS) and Marie Stopes International/Ghana (MSIG), as well as the United States Agency for International Development (USAID)/Ghana health team, have identified access to implant removal as an important element of family planning program strengthening.

We designed this study to generate evidence on the state of access to implant removal services in Ghana and to identify areas for improvement that could inform programs globally.

The modern contraceptive prevalence rate among married users in Ghana has nearly doubled in the past few years from 18.4% in 2013 to 30.7% in 2017 with implants making up 2.9% and 8.4%, respectively, of the modern contraceptive prevalence rate.^9^^,^^10^ In 2013, 15.7% of married users were using implants^9^ and by 2017, this had increased to more than 30%,^10^ making implants the most commonly used method in the country. Implant use is also common among unmarried women with 17.8% of contraceptive users reporting implant use in the most recent survey.^10^ Use is widespread across the country, and in the last 5 years, Ghana has procured more than 1.3 million implants.^8^ In 2013, as part of a government effort to expand access to contraceptive implants, GHS revised its policy on implant provision, allowing community health nurses to insert and remove contraceptive implants.^11^ In addition, access to contraceptive implants has been promoted through the outreach services of private organizations such as MSIG, which has provided implants in its mobile outreach services at GHS facilities in Central and Western regions since 2011. Implants are an affordable long-acting and reversible method in Ghana offered to women for 2 Ghana cedis (42 US cents) in the public sector (inclusive of removal) and free of charge through MSIG outreach.

Our study had 4 objectives: (1) to measure implant acceptors’ knowledge of the possibility of removal before labeled duration of use and when and where to obtain removal; (2) to describe reasons for seeking removal; (3) to estimate the proportion of implant acceptors who were able to get their implant removed; and (4) to document barriers to removal. The study was implemented by FHI 360 in collaboration with the Population Council of Ghana, MSIG, and GHS.

## METHODS

### Study Design

We conducted a mixed-methods study using a retrospective design to examine the removal desires and experiences of women who had received an implant, as well as the experiences of family planning providers. To capture dynamics surrounding access to implant removal services in different service delivery contexts while also ensuring some geographic and sociocultural diversity, we conducted the study in 2 regions with public sector service delivery through GHS facilities (Ashanti and Eastern regions) and 2 regions with MSIG mobile outreach services (Central and Western regions). The study was not designed to support comparison between the 2 service delivery contexts, but rather to inform recommendations for potential strengthening of services on a larger scale to benefit a greater number of women. To reach large numbers of women and identify those who wanted to have their implant removed, we conducted a phone survey with implant acceptors and an in-person exit survey with women receiving implant removal services from mobile teams (outreach regions only). In addition, we conducted follow-up in-depth interviews (IDIs) with a subset of implant acceptors to obtain a more detailed understanding of the circumstances affecting women’s ability to obtain removals to determine concrete ways to improve access, and IDIs with providers to provide detailed insights into possible constraints on the service delivery side. More detailed methods and study results are available from the programmatic report published online.

FHI 360’s Protection of Human Subjects Committee, the Ghana Health Service Ethical Review Committee, and the Marie Stopes International Ethical Review Committee approved this study. Phone survey participants provided oral consent and in-person exit survey and IDI participants provided written consent.

### Study Populations and Sample

#### Phone Survey With Implant Acceptors

In the public sector, a mobile and web-based system known as the reproductive services log (rsLog) electronically captures family planning and reproductive health data from clinic registers. The rsLog has been in use since January 2015 and was operational in 95 GHS facilities in Ashanti and Eastern Regions (approximately 30% of the public sector facilities in the 2 regions) at the time the study was conducted. Similarly, MSIG implemented its electronic client information center database in 2014 that includes service delivery data from outreach and static clinic clients. We focused our sample on women included in these electronic records. Eligible women for the phone survey included those who: (1) had an implant inserted between 1 January 2015 and 31 December 2016 at a GHS facility in public regions or between 1 July 2014 and 31 December 2016 through MSIG mobile teams in outreach regions, (2) were aged 18–49 years at the time of implant insertion, and (3) had phone information available in their records.

Our sampling approach aimed to complete a minimum of 384 interviews of women who had ever wanted a removal in each context (public and outreach). We computed our sample size to be able to estimate the proportion of implant acceptors who were able to get their implant removed at first attempt with 95% confidence and 5% precision. Sample size calculations also assumed a base estimate of 50% as there was no preliminary information about the indicator of interest and to be conservative for sample size purposes.

**FIGURE 1.**
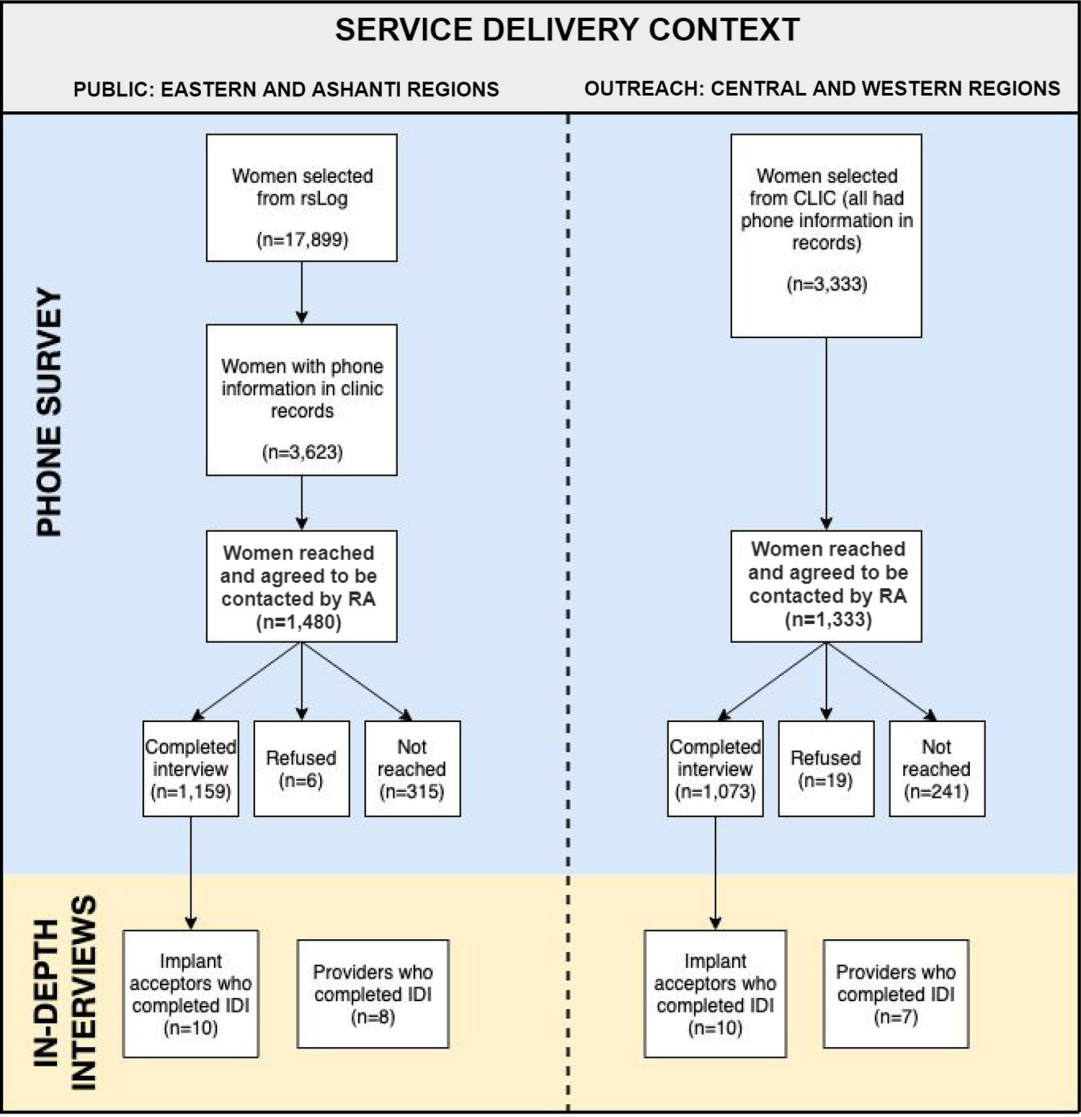
Study Sample of Women, Aged 18–49 Years Old, Who Had a Contraceptive Implant Inserted in Public and Outreach Regions, Ghana Abbreviations: CLIC, Client Information Center; IDI, in-depth interview; RA, research assistant; rsLog, Reproductive Services Log.

In public regions, we selected a random sample of women stratified by health facility from the rsLog. Given that phone access information was not readily available in the rsLog, we oversampled women from the rsLog, looked for phone information in their clinic records, and proceeded to call those with phones. In outreach regions, we attempted to recruit all women in the electronic client information center database who met study eligibility criteria as the target sample size was not expected to be met unless all women were recruited. Women in both settings were first contacted by phone by GHS facility or MSIG staff and asked whether they would be willing to be contacted by a researcher associated with the study (Figure 1).

#### Exit Interview With Women Receiving an Implant Removal

For practical reasons related to the longer duration of the program and preliminary information on the volume of clients seeking removal services, exit interviews were only implemented in the outreach context. We interviewed a convenience sample of women aged 18–49 years obtaining removal services from MSIG mobile teams to obtain information from women who had used an implant for longer periods. Trained research assistants were deployed at mobile outreach sites on discrete days and recruited women as they exited. Specific outings were selected based on convenience according to scheduled activities. MSIG providers asked all eligible women whether they would be willing to participate in the survey and study staff interviewed women in a private location after removal. Data collection was conducted during outreach outings until an overall target of 50 interviews were complete. The sample size was determined by budget and logistic considerations.

#### IDIs with Implant Acceptors

We conducted IDIs with a subset of women (n=20) who participated in the phone survey and provided permission to be contacted again. IDIs were conducted in 1 public (Eastern Region) and 1 outreach region (Central Region) only for practical considerations. The participants represented 4 different profiles: (1) women who obtained removal at first attempt, (2) women who obtained removal on second or subsequent attempt (referred to as “delayed success”), (3) women who made at least 1 removal attempt but had not yet obtained removal by the time of the study, and (4) women who reported wanting removal but had not yet tried to access removal services. Sample size targets were set to achieve a minimum of 4 IDIs per target profile, influenced by a combination of time and budget constraints and general acceptance that 4–5 IDIs are sufficient to identify the most important themes of a study. Recent evidence indicates that 80% saturation can be reached within 8 IDIs; we aimed for 8 IDIs for the main group of women with delayed removals (Figure 1).^12^

#### IDIs With Family Planning Providers

We also conducted IDIs with a convenience sample of family planning providers (n=15) who conduct implant insertions and removals at GHS facilities in public regions (GHS providers) and at GHS facilities partnering with MSIG in outreach regions (GHS and MSIG providers) (Figure 1).

### Data Collection and Analysis

#### Phone and Exit Survey

Trained data collectors conducted phone and in-person exit interviews between October 2017 and January 2018 in either English, Twi, or Fante, according to the participant’s preference. Respondents to the phone survey were compensated 5 Ghana cedis (approximately US$1) in mobile money pushed to their device; and exit interview respondents were provided the same amount in cash at the time of the interview. At the time of the interview, data collectors entered data into tablets containing an Open Data Kit survey program. Data were transferred from the tablets to a secure server immediately using a cellular network or wireless connection. Data were then deleted from the tablets to ensure participant confidentiality. Data collection forms were programmed with automatic constraints to minimize human error. Data collector supervisors performed spot quality checks on the data and an analyst at FHI 360 in North Carolina conducted additional quality checks.

We analyzed data by study objective separately for each study context (public and outreach). For women who attempted removal, we classified removal by timing of attempt: first attempt, within 1 week of first attempt, longer than 1 week after first attempt, and attempted but not yet removed. We further divided unsuccessful removal attempts by stated desire to keep or remove the implant in the future.

We calculated sampling weights for the public sector sample as the inverse of the probability of selection of implant clients for the phone interviews. Weights were calculated based on the number of implant insertions among women of eligible age during the study eligibility period at each facility as per the rsLog and the total number of interviews completed per facility. All analyses (e.g., percentages, means, standard deviations, and ranges) were weighted; however, we present unweighted frequencies to clearly indicate the available sample size. Results are presented descriptively except for the indicators associated with seeking removal, for which we provide 95% confidence intervals (CIs) for the public region data. The relative wealth of all implant acceptor study participants was calculated using a subset of variables from the USAID Poverty Assessment Tool,^13^ though as all variables from the tool were not included in the survey, we were unable to categorize respondents as below or above the poverty line in Ghana, as is the intent of the tool. Rather, we used principal components analysis to create a score representing a relative measure of wealth within the sample. We used STATA version 13 (StataCorp, College Station, TX) for all analyses.

#### IDIs

Two trained qualitative interviewers conducted the IDIs with women in the language of their preference (English, Twi, or Fante) and providers in English between November 2017 and July 2018 using topic guides. The interviewers audiorecorded all interviews and transcribed them into English. The study team developed codebooks, which included both inductive and deductive codes. Two team members coded the transcripts using NVivo 11 (QSR International, Burlington, MA) and incorporated periodic intercoder reliability checks of 15% of transcripts to ensure coding consistency. The team developed detailed thematic memos to identify key dimensions of each code and synthesize data. Women who participated in IDIs were compensated 5 Ghana cedis (approximately US$1) for their time and reimbursed for travel expenses to the interview site. Providers did not receive compensation for participating in the study as the interviews occurred at their workplace during regular work hours.

## RESULTS

### Phone Survey Results

#### Study Population and Participant Characteristics

In the public context, 1,159 women participated in the survey, representing a 78.3% response rate of women who agreed to be contacted by a study research assistant. In the outreach context, 1,333 women participated in the study with an 80.5% response rate (Figure 1). Survey participants were, on average, aged 28.4–29.6 years, married or cohabitating, and had 2.3–2.6 children (Table 1). Most participants had at least some primary education, and in the public regions, 30.6% had a high school or higher education, while in the outreach regions one-fifth had a high school or higher education.

**TABLE 1.** Contraceptive Implant Acceptor Phone Survey Participants’ Demographic Characteristics by Context, Ghana

	**Public** ^a^ **(N=1,159)**	**Outreach** **(N=1,073)**
Age, years, mean (SD)	29.6 (6.6)	28.4 (6.2)
18–29, %	53.8	63.1
30–39, %	36.8	30.7
40–49, %	9.4	6.2
Marital status		
Never married, %	21.4	19.5
Married/cohabitating, %	73.0	76.4
Divorced/widowed, %	5.6	4.2
Parity	(n=1152)	(n=1062)
Mean (SD)	2.3 (1.6)	2.6 (1.6)
0, %	10.2	3.9
1–2, %	49.0	50.1
3–4, %	31.5	33.3
5+, %	9.4	12.7
Highest education		(n=1072)
None, %	6.7	5.5
Primary, %	11.0	15.5
Middle, %	51.8	58.4
High school, %	21.7	15.9
>High school, %	8.9	4.7
Religion		
Christian, %	92.3	93.6
Muslim, %	7.4	4.9
Other/none, %	0.3	1.5
Have health insurance, %	64.7	42.6
Wealth quantiles	(n=1155)	(n=1073)
Lowest, %	16.3	23.9
Second, %	18.2	22.1
Middle, %	21.1	20.3
Fourth, %	22.4	18.0
Highest, %	22.0	15.8
Months since implant inserted		
Mean (SD)	19.5 (10.8)	14.5 (7.4)
0–6, %	4.9	12.6
7–12, %	34.0	46.3
13–18, %	19.4	13.4
19–24, %	18.7	22.4
25–36, %	18.9	4.9
>36 months, %	4.1	0.5
Implant type^b^		
Jadelle, %	61.9	86.3
Implanon, %	29.8	6.3
Unknown, %	8.3	7.4

Abbreviations: SD, standard deviation.

a Frequencies are unadjusted; percentages and means are adjusted for sampling weights.

b Implant type determined by comparing participant responses to number of rods in their implant and the duration of protection. Response combinations that do not describe any available implant are categorized as unknown.

The majority of women surveyed in the public and outreach contexts had their implants inserted for less than the labeled duration of use with only 4.1% of women in the public context and less than 1% in the outreach context reporting having their implant inserted more than 3 years ago (Table 1). Most women (89.5% in public and 93.7% in outreach context) did not know the name of their implant; however, most knew the number of rods and its duration of protection. Using this information, we estimated that 61.9% of women in the public context and 86.3% in outreach had Jadelle. Between 7.4%–8.3% of respondents did not provide a response combination that described any available implant; thus, we could not determine their implant type (Table 1).

#### Knowledge of When and Where to Obtain a Removal

Most women (88.2% [95% CI=85.8, 90.3] in the public context and 84.3% in outreach) were aware that their implant could be removed before its labeled duration. Most of these women said that their provider gave them this information: 88.9% (95% CI=86.0, 91.3) and 88.4% in the 2 contexts, respectively. Across contexts, desire for pregnancy and experience of side effects were the most common reasons women reported that their providers told them that an early removal was possible. Although approximately two-fifths of women in both settings said that their provider told them of at least 2 places where they could access to removal, nearly half of study participants in the public context and one-third of outreach respondents reported that their provider told them that they could have their implant removed only at the site where they had it inserted. For outreach clients, interviewers clarified that the location of insertion referred to the outreach services (Table 2).

**TABLE 2.** Phone Survey Participants’ Reported Knowledge of Contraceptive Implant Removal Services by Context, Ghana

	**Public** ^a^ **(N=1,159)**		**Outreach** **(N=1,073)**
	**%**	**95% CI**	**%**
Aware implant can be removed before labeled duration	88.2	85.8, 90.3	84.3
Told by provider at insertion that implant can be removed before labeled duration	(n=1020) 88.9	86.0, 91.3	(n=905) 88.4
Reasons provider mentioned that implant can be removed before labeled duration^b^	(n=923)		(n=800)
Want children	69.3	N/A	62.6
Side effects	60.5	N/A	62.0
Any reason	26.0	N/A	24.9
Partner disapproves	9.9	N/A	9.5
Told at insertion where removal can be obtained			
Insertion place only	46.5	N/A	33.3
Place other than insertion place	3.2	N/A	15.2
Insertion place and another place	40.5	N/A	38.1
Not told about any place/don’t know	9.8	N/A	13.4

a Frequencies are unadjusted; percentages and means are adjusted for sampling weights.

b Multiple responses possible, spontaneous mention.

#### Reasons for Seeking Removal

Approximately one-third of women in the public sector and one-fifth in the outreach sector reported ever wanting to have their implant removed regardless of whether they attempted removal. The main reason reported for wanting a removal was experience of side effects, including menstrual bleeding side effects, and health concerns (62.1% in public sector, 72.3% in outreach) (Table 3).

**TABLE 3.** Phone Survey Participant’s Reported Desire to Remove Contraceptive Implant, by Context, Ghana

	**Public** ^a^ **(N=1,159)**	**Outreach** **(N=1,073)**
	**%**	**%**
Report wanting removal	31.8	21.5
Main reason for wanting removal/obtaining removal	(n=373)	(n=231)
Other side effects/ health concerns	37.7	49.4
Bleeding side effects	24.4	22.9
Wanted children	18.7	10.4
Partner disapproved	4.9	5.2
Lost partner/partner away/infrequent sex	3.5	2.6
Other	2.7	4.8
Implant expired	2.5	0.0
Afraid of becoming infertile	2.2	1.3
Sexual side effects	1.4	1.3
Became pregnant	1.1	1.3
Too old/ menopause/ infecund	0.7	0.4
Don’t know	0.2	0.4

a Frequencies are unadjusted; percentages and means are adjusted for sampling weights.

Table 4 shows women’s reports of side effects with use of their implant and influence by others to remove their implant. Among participants who ever wanted their implant removed, 85.2% and 90.0% in the public and outreach sectors, respectively, reported experiencing bleeding side effects. About three-quarters across both contexts who did not desire removal reported experiencing bleeding side effects. Regardless of removal desire and across contexts, the most common bleeding side effect was amenorrhea. Similarly, more women who wanted a removal, versus those who did not, reported experiencing nonmenstrual-related side effects: 59.6% versus 40.3% in the public context and 70.0% versus 38.0% in the outreach context. Dizziness and weight change were the most commonly reported nonmenstrual-related side effects.

**TABLE 4.** Phone Survey Participants’ Reported Contraceptive Implant Side Effects and Social Influence by Context and Desire to Remove Implant, Ghana

	**Public** ^a^ **(N=1,159)**		**Outreach** **(N=1,073)**	
	**Ever Wanted Removal** **(n=373)** **%**	**Never Wanted Removal** **(n=786)** **%**	**Ever Wanted Removal** **(n=231)** **%**	**Never Wanted Removal** **(n=842)** **%**
Reported experiencing bleeding side effects	85.2	75.6	(n=230) 90.0	(n=840) 77.3
Most commonly mentioned bleeding side effects^b^	(n=315)	(n=597)	(n=208)	(n=651)
Stopped having period	39.3	37.2	46.6	48.9
Bleed more during period	37.6	25.7	19.7	14.8
Bleed less during period	23.8	25.5	22.1	24.3
Period lasts longer	26.0	23.0	26.4	24.3
Period is shorter	13.8	15.4	18.3	18.0
Reported experiencing side effects (other than bleeding)	(n=372) 59.6	(n=784) 40.3	(n=230) 70.0	(n=840) 38.0
Most commonly mentioned other side effects^b^	(n=227)	(n=315)	(n=161)	(n=320)
Dizziness	47.1	26.5	50.3	38.4
Weight change	43.2	40.1	40.4	33.4
Headaches	23.8	26.4	15.5	13.1
Abdominal pain	11.1	21.9	14.9	15.9
Reported someone influenced to stop using implant	40.5	31.8	51.5	29.8
Person(s) influenced by^b^	(n=154)	(n=237)	(n=118)	(n=215)
Neighbor or friend	54.9	83.5	54.2	76.9
Husband or partner	42.1	14.4	31.4	12.0
Mother	9.7	7.2	14.4	6.8
Other person/unspecified	3.8	9.9	10.2	10.8

a Frequencies are unadjusted, percentages and means are adjusted for sampling weights.

b Multiple responses possible, spontaneous mention.

In terms of social influence to remove implants, 40.5% and 31.8% of women in the public context who wanted and did not want a removal, respectively, reported being influenced by someone to remove their implant. Most commonly, this was a neighbor/friend followed by a husband/partner. In the outreach sector, more social influence (51.5%) was reported among women who wanted a removal versus women who did not want a removal (29.8%) with the most commonly mentioned influence being a neighbor or friend (Table 4).

#### Experience Seeking Removal

**FIGURE 2.**
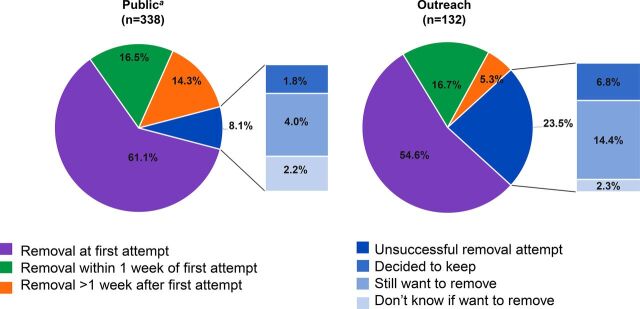
Outcomes Among Women Who Attempted to Have Contraceptive Implants Removed in Public and Outreach Regions, Ghana ^a^Percentages are adjusted for sampling weights.

The majority of women in the public sector who reported ever wanting a removal also sought removal (91.9% [95% CI=86.7, 95.1]) (data not shown). Of these, 61.1% (95% CI=54.8, 67.1) obtained a removal at their first attempt and an additional 16.5% (95% CI=12.2, 21.9) obtained a removal within 1 week of first attempt. At the time of the survey, 8.1% (95% CI=5.1, 13.6) of women wanting a removal had not yet had their implant removed despite attempting at least 1 time. Of these women, 4.0% (95% CI=2.3, 6.9) decided that they still desired to remove the implant, 2.2% (95% CI=0.5, 9.8) were unsure if they still desired to remove the implant, and 1.8% (95% CI=0.9, 3.7) decided that they no longer desired to remove the implant (Figure 2).

In the outreach sector, 57.1% of women who reported ever wanting a removal attempted to have their implant removed (data not shown) and just over half of these women (54.6%) obtained removal on their first attempt. An additional 16.7% were able to get a removal within a week of their first attempt. Of the women who wanted a removal but never attempted removal, the top stated reasons were changing their mind (37.5%), too expensive (16.7%), and being too busy (10.4%) (data not shown). Approximately one-quarter of women had not yet received a removal despite attempting at least once at the time of survey (Figure 2).

Most women tried obtaining a removal from the same place they received their implant (80.5% public, 71.8% outreach) for their first attempt. As described above, for outreach clients “same place” referred to outreach. Reasons for going to a different place for the first removal attempt included distance, scheduling inconvenience, and cost. Of women who made more than 1 attempt to obtain a removal, the majority returned to the same facility for all attempts (89.2% in public, 78.4% in outreach). A minority of women visited 2 separate facilities (10.8% in public, 18.9% in outreach) and 2.7% of women in the outreach sector visited 3–5 facilities to obtain a removal (data not shown).

#### Barriers to Removal

Women who attempted to have a removal were asked about potential barriers they faced. In the public context, approximately one-fifth reported keeping their implant because a provider counseled to continue using. In the outreach context, provider counseling to continue using and provider unavailability were each reported by 16.7% of women attempting a removal. Just over 10.0% in both contexts reported that there was a time that the provider would not remove their implant though the woman wanted a removal. In the outreach context, 13.5% said that the provider could not palpate their implant and, thus, could not remove. Women also reported complications they experienced with implant removal. Approximately 40.0%–50.0% experienced temporary pain at the time of removal and/or pain that lasted a few days after the removal. In the outreach context, over one-fifth of women reported having continued pain for more than a few days, and 5.2% of women mentioned the same in the public context. Overall, most women reported that their entire experience getting their implant removal—from the time they decided to get a removal until the time they got a removal—was very or somewhat easy (73.8% in public, 68.4% in outreach) (Table 5).

**TABLE 5.** Phone Survey Participants’ Responses on Barriers to Contraceptive Implant Removal and Satisfaction With Services Among Women Who Attempted a Removal by Context, Ghana

	**Public** ^a^ **(n=339)**	**Outreach** **(n=132)**
	**%**	**%**
Reason women reported they could not get a removal when they wanted to^b^		
Provider counseled to continue using	20.6	16.7
Provider not available	8.6	16.7
Provider would not remove	11.5	10.6
Provider unable to remove despite trying	0.7	4.6
Implant not palpable	4.4	13.5
Problems at removal site on arm^c^	(n=314)	(n=101)
Temporary pain at time of removal	43.6	46.0
Pain that lasted a few days	43.6	54.1
Scarring	35.2	35.1
Infection/swelling	5.1	5.4
Continue pain	5.2	21.6
Other unspecified	0.0	5.4
Ease of removal experience (among women who had a removal)	(n=314)	(n=101)
Very easy	53.0	55.5
Somewhat easy	20.8	12.9
Somewhat difficult	17.4	16.8
Very difficult	8.8	14.9

a Frequencies are unadjusted; percentages and means are adjusted for sampling weights.

b Each of these options were asked as a separate yes/no question.

c Multiple responses possible, spontaneous mention.

Most women who received an implant removal reported paying for the service (75.0% in public, 84.2% in outreach). The average cost women reported for removal services was 15.2–24.6 Ghana cedis (US$3.20–$5.00) depending on the service delivery context. Women in both the public and outreach sectors also reported spending, on average, 5.2 Ghana cedis (US$1.10) for transportation to and from the removal procedure location (Table 6).

**TABLE 6.** Phone Survey Participants’ Reported Costs Associated With Contraceptive Implant Removal Services Reported by Respondents Who Removed Implant by Context, Ghana

	**Public**^a^(n=307)	**Outreach**(n=101)
Reported incurring cost for removal services not associated with transportation^b^, %	75.0	84.2
Mean cost incurred, US$^c^ (SD)	3.2 (1.8)	5.0 (1.9)^d^
Reported incurring cost for transportation, %	70.6	55.5
Mean cost incurred, US$^c^ (SD)	1.1 (0.7)	1.1 (1.0)

Abbreviation: SD, standard deviation.

a Frequencies are unadjusted; percentages and means are adjusted for sampling weights.

b Costs includes fees for supplies, provider, and other facility-associated costs.

c Respondents reported costs in Ghana cedis. Costs were converted to US$ using the exchange rate at the time of analysis (21 US cents=1.00 Ghana cedi). Means calculated from women who reported incurring costs only.

d One respondent reported a cost of US$52.50, which was an extreme outlier. This response was removed from the analysis.

#### Method Uptake and Unmet Need for Family Planning After Removal

Approximately one-third of women across contexts who had their implant removed reported adopting a different method of family planning either at the time of removal or later. Most of these women started using the injectable. Women who did not adopt a new method cited pregnancy desire (37.4% in public, 18.1% in outreach) and side effects (34.8% in public, 36.1% in outreach) as top reasons. Unmet need, defined as not adopting a method of FP after removal for all reasons other than desiring pregnancy, reporting no sexual activity, or infecundity, was 33.3% in public and 53.5% in outreach (Table 7).

**TABLE 7.** Phone Survey Participants’ Reported Contraceptive Method Uptake and Unmet Need After Implant Removal by Context, Ghana

	Public^a^(n=314)	Outreach(n=101)
	%	%
Adopted contraceptive method after removal	35.6	28.7
Method used	(n=116)	(n=29)
Injectable	60.2	72.4
Pill	24.8	24.1
Emergency Contraception	11.1	3.5
Intrauterine device	2.4	0.0
Implant	0.7	0.0
Other	0.8	0.0
Reasons for not adopting method	(n=193^b^)	(n=72)
Want to get pregnant	37.4	18.1
Side effects/health concerns	34.8	36.1
Lost partner/partner away	11.2	6.9
Partner disapproves	3.9	11.1
Inconvenient	1.3	4.2
Other/unspecified	11.4	23.8
Unmet need for family planning after removal^c^	33.3	53.5

a Frequencies are unadjusted, percentages and means are adjusted for sampling weights.

b Five respondents did not provide a response to this question, thus the reduced sample size.

c Unmet need is defined as not adopting a method of family planning after removal for all reasons other than desiring pregnancy, reporting no sexual activity, and reporting infecundity.

### Exit Survey Results

Fifty women completed the exit survey: 34 in Central Region and 16 in Western Region. Average age was 31.0 years, and more than half were married (58.0%) and had an average of 3.3 children. The participants in the exit survey were notably less wealthy than phone survey respondents, with 55% falling into the lowest wealth quintile and no participants representing the highest wealth quintile. Most women had Jadelle (84.0%) and were getting a removal due to the implant expiring, with two-thirds of women reporting having had their implant at least 60 months. All but 1 of the women interviewed received their implant removal on the first attempt (data not shown) and two-thirds adopted a method immediately, with most receiving a new implant (93.9%) (Table 8).

**TABLE 8.** Characteristics of Contraceptive Implant Acceptor Exit Interview Participants, Ghana

	**Interviewees**(n=50)
Age, years, mean (SD)	31.0 (7.0)
Marital status, %	
Never married	30.0
Married/cohabitating	58.0
Divorced/widowed	12.0
Parity, mean (SD)	3.3 (1.8) (n=47)
Highest education, %	
None	22.0
Primary	36.0
Middle	42.0
Religion, %	
Christian	92.0
Muslim	6.0
Other/none	2.0
Have health insurance, %	16.0
Wealth quantiles, %	(n=49)
Lowest	55.1
Second	28.6
Middle	12.2
Fourth	4.1
Highest	0.0
Months since implant inserted, %	(n=48)
Mean (SD)	51.4 (16.8)
≤ 24	12.5
25–36	12.5
48	8.3
60+	66.6
Implant type^a^, %	
Jadelle	84.0
Implanon	4.0
Unknown	12.0
Adopted contraceptive method after removal, %	66.0
Contraceptive method used, %	(n=33)
Intrauterine device	3.0
Implant	93.9
Female sterilization	3.0
Reasons for not adopting method, %	(n=16^b^)
Want to get pregnant	25.0
Side effects/health concerns	12.6
Partner disapproves	31.3
Inconvenient	18.8
Other/unspecified	12.6
Unmet need for family planning after removal^c^	24.0

a Implant type determined by comparing participant responses to number of rods in their implant and the duration of protection. Response combinations that do not describe any available implant are categorized as unknown.

b One respondent did not provide a response to this question, thus the reduced sample size.

c Unmet need is defined as not adopting a contraceptive method after removal for all reasons other than desiring pregnancy, reporting no sexual activity, and reporting infecundity.

### Qualitative Results

#### Client Study Population and Participant Characteristics

A subset of 20 implant acceptors from the phone survey, evenly divided between the public and outreach contexts, participated in IDIs. Five had received a removal at first attempt, while 10 received a removal at second or subsequent attempt and 2 participants wanted a removal but had not obtained a removal at the time of the interview despite having tried. Three women in the outreach context had wanted a removal but never attempted to get a removal. (This situation was not explored in the public context as it was not commonly reported in the quantitative data) (Table 9).

**TABLE 9.** Outcome of Either Removal Attempt or Desire to Remove for Contraceptive Implant Acceptor In-depth Interview Participants by Context, Ghana

	**Public**(n=10)	**Outreach**(n=10)
	No.	No.
Successful removal at first attempt	3	2
Removal at second or subsequent attempt	6	4
Removal not yet obtained	1	1
Wanted removal but have not attempted	0	3

#### Reasons for Wanting Removal

Qualitative IDIs revealed that women’s reasons for seeking removal were often multifaceted. Apart from 2 women who chose to remove their implants because they wanted to become pregnant, all women cited more than 1 reason for wanting a removal. Most of the women interviewed said that bleeding changes, including prolonged bleeding, irregular bleeding, and amenorrhea, contributed to their decision to seek removal. Several women also reported that other side effects contributed to their desire for removal. Changes in weight were frequently mentioned, though no participants stated that weight change was the only reason for removing:

*As for me, the particular reason I wanted it removed is the way my heart was troubling me and the way I felt pain in my abdomen, coupled with how I grew lean. I grew lean and became very smallish and my veins were visible. I grew lean.* —Woman, 22 years old, has 1 child

As the quote above demonstrates, women often experienced a variety of side effects that, taken together, led them to want a removal. Other commonly reported side effects that influenced removal decisions included headaches and abnormal heartbeat or heart palpitations, and a few women reported dizziness or nausea. Some women reported that the side effects they experienced interfered with their daily lives or made them unable to work for some period of time:

*When it* [heart pain] *was happening, I couldn’t work for a week. I will be at home all week long.* —Woman, 42 years old, has 7 children

About half of the women who participated in IDIs also described their partner as playing a role in their decision to seek a removal. Most of these women said their partners wanted them to get a removal due to changes in bleeding or other side effects that they worried were harming their health. Seven women stated that their partners “told” or “forced” them to get a removal:

*… The last implant he forced me to remove I had not removed the plaster when I visited him, so he saw it fresh and since then he was always on my neck to remove it until I got it removed after just 1 month of insertion. Left to me alone, I would have waited another 5 years because I had 3 children then.* —Woman, 34 years old, has 4 children

In contrast, a few women reported that their partners either did not want the removal or supported their own decision to remove without exerting pressure:

*I wanted to have it removed and inject the 3 months one. And he said, “Ok, that will be fine”.* —Woman, 22 years old, has no children

Apart from partners influencing women to get a removal, 4 women reported that their mothers encouraged them to remove their implants.

#### Removal Experience

Nearly half of all clients in both contexts reported that an early removal was more expensive than removal at labeled duration:

*Yes, he [the provider] explained that the removal procedure was generally free at due date, but I was charged because I was removing it before the due date.* —Woman, 37 years old, has 3 children

About one-quarter of the women, particularly in the outreach context, expressed that cost limits women’s access to removal. Some women in the outreach context explained that it is often permanent public facility staff who do removals, rather than MSIG outreach workers, and that there is a cost associated with the removal in this case.

*The same facility that I went for the free family planning services but those people who came to do it for free for the community were not around but the regular service providers at the clinic were still here and they told us on the day of insertion that we can come for removal at a cost of 30 [Ghana cedis] anytime we want to remove it.* —Woman, 34 years old, has 4 children

Of the 12 women who had visited a provider at least once but still had their implant, most reported being told to come back on a different day. A few of these women were told that the provider who could remove implants was not available on the day they visited or that they should go to the facility where their implant was inserted. Several of these women who were experiencing bleeding changes reported being given medication to treat heavy bleeding or were counseled that their bleeding changes were normal.

All women who told providers they were planning to get pregnant or that their husbands/partners did not approve of their use of the implant were successful in obtaining removal on their first attempt:

*I pleaded with them to remove it for me because my husband didn’t want me to do it and so I didn’t inform him before I inserted it. He wasn’t in agreement for me to do it at all. We even had a quarrel at home because of that….I told them that I didn’t inform my husband. And so he is angry because I didn’t inform him. I also had some side effects that he is spending money on and so he is always complaining and so they should remove it for me. I told them I was scared of my sight and urinating problems and I was also scared that my husband will stop taking care of me and leave me and so I am pleading with them they should remove it for me. I wept in front of them.* —Woman, 38 years old, has 3 children

Women also reported obtaining a removal successfully when they told their providers about ongoing heavy bleeding or other severe side effects persisting despite treatment, hearing complaints from family members at the facility that the woman was denied a removal, and agreeing to begin another method of family planning. Many women felt that providers only agreed to perform the removal reluctantly:

*They were trying to convince me not to remove it. But since I already made up my mind, they agreed to do it for me.* —Woman, 27 years old, has no children

About half of the women who received removals, particularly in the public context, reported that the removal procedure was easy or not painful; however, nearly the same number of women, but more evenly split between the public and outreach contexts, reported that removal was difficult, painful, or took a long time. Four women (2 in each context) experienced broken rods, deep removals, or nonpalpable removals.

#### Provider Study Population and Participant Characteristics

Fifteen providers, 8 in the public context and 7 in the outreach context, participated in IDIs. Seven were community health nurses, 4 were midwives, and 2 were nurses; 2 providers in the public context held other positions. Most providers in the public context had between 1 and 2 years of experience both inserting and removing implants, and most in the outreach context had between 3 and 5 years of experience. One provider in the public context had no experience with implant removals. Of the providers from the outreach context, 3 were MSIG staff members; the rest were GHS providers working in facilities where MSIG conducts outreach (Table 10).

**TABLE 10.** Characteristics of Contraceptive Implant Providers by Context, Ghana

	**Public** **(n=8)**	**Outreach** **(n=7)**
	**No.**	**No.**
Provider cadre		
Nurse	1	1
Midwife	2	2
Community health nurse	3	4
Other	2	0
Experience with implant insertion		
1–2 years	5	1
3–5 years	0	5
6+ years	3	1
Experience with implant removals		
No experience	1	0
1–2 years	4	3
3–5 years	0	4
6+ years	3	0

#### Provider Perspectives

Most providers who participated in IDIs stated that they told women during initial counseling that they could have implants removed before the expiration date; of these, many stated they counseled women that they could have their implant removed if they wished to conceive, while others said they told women they could remove the implant at any time and for any reason.

*With the implant, what I normally do is, when I insert it for you, I tell them it’s not because they’ve stated 3 years, you should use it for 3 years. If you want to conceive in a year or 2, you can come for your removal. That is what I normally tell them. —*Community health nurse with 1 year of experience providing implants

Nearly all providers expressed that early removals were appropriate in the case of severe side effects such as excessive bleeding, heart palpitations, or high blood pressure, but almost half said they always tried to treat side effects before removing an implant:

*First of all, you try to counsel the client upon what reason the person gave. But when you come the first day, we wouldn’t do it for you the first day. If it’s bleeding, we will try the ibuprofen or the microgynon and then if still you go and come back and say it didn’t help you, so still you want to remove, then we do it for you.* —Disease control officer with 7 years of experience providing implants

Two-thirds of providers described the importance of counseling when women came for removal before the labeled duration. Some providers stated that potential side effects may not have been properly explained at insertion, leading women to blame an implant for unrelated issues. Most providers described demand by a woman’s husband or partner as an acceptable reason for removal. About half of the providers felt it was appropriate to do an early removal if a client wished to conceive. However, a few in the outreach context said they decided whether or not a client was “ready” to have another child before removing the implant. A few providers also stated that they decided whether or not a client’s side effects were severe enough or enough time had elapsed since insertion before agreeing to remove:

*When asked why [the client wanted a removal], she said, “I don’t experience my menses and because of that my boyfriend has left me and so I want to remove it. But I am not worried because he left me, but I am worried because I don’t experience my menses as a result of the implant.” And I said, “I won’t remove it for you. Because it’s just 3 months, I can’t take it off for you.”* —Community health nurse with 1 year of experience providing implants

Providers reported a range of costs for implant removal services, and a few reported that they charged more for a removal before the implant’s expiration. Several providers felt that most women were able to pay the removal fee, though more said they were, at times, willing to perform removals for less than their normal fee if the woman could not pay.

Most providers, including all 7 from the public context, reported experiences with removal of broken or bent rods or rod fragments. Many felt that the frequency with which they encountered difficult removals was due to poor insertion by other providers, including implants being inserted into muscles or the wrong part of the body. Nearly half of providers said they had done removals that were painful for the client, and some providers noted that these painful removals could discourage implant use in communities. More than half of the 15 providers reported ever having made a referral to a different facility or seeking assistance from other facility staff for difficult removals. Several providers mentioned using or referring for x-rays, “scans,” or MRIs to detect implants for removal.

#### Provider Needs

Although nearly all providers indicated they felt confident removing implants due to training and experience, about one-third of providers, particularly in the public context, reported they did not feel confident with difficult removals. Most providers stated they would welcome—or in some cases needed—additional training, with an equal number specifically mentioning additional training on counseling and removal:

*With removal, it involves a lot … Going into the skin, if you don’t take care you might even infect it. And then if the insertion is too deep…you might even be damaging some veins, which can lead to blood loss or other things. At least if I’m able to get the training, at least I will get the necessary skills that I will be able to do the removal without damaging any tissue or any of this thing.* —Registered community nurse with 2 years of experience providing implants

Apart from training, nearly all providers expressed a need for additional equipment or instruments to perform removals, specifically forceps and autoclaves or other sterilization equipment.

## DISCUSSION

This study represents one of the few systematic assessments of contraceptive implant users’ access to implant removal in a developing country setting.^3^^,^^14^ Encouragingly, we found that a large majority of implant users in both public and outreach settings knew when and where to obtain removal and that more than half of users who had attempted removal were able to get their implant removed on first attempt with an additional 20%–30% on a subsequent attempt. It is important to note that our measure of “success” in accessing removal is crude and does not capture experiences where women may have received counseling that led them to decide to keep their implant. Future research should take into account satisfaction with such provider interactions.

Removal knowledge and access were not universal, however, and many women reported having only been told that they could have their implant removed where they had received it. We also found that more than half of women who had obtained an implant from an MSIG outreach provider and said they wanted a removal did not actually attempt to have their implant removed. Some of these women reported that they changed their mind about wanting their implant removed, and others indicated that cost and the need to travel to a facility presented barriers.

In addition to describing access to removal services, this study also provides some insight into why implant users seek removal before expiration. Our survey results indicate that side effects, including menstrual bleeding changes, were the predominant reason for desiring early removal. However, the qualitative findings provided a more complete picture, showing that the decision to remove “early” was often multifaceted. Either a combination of different kinds of side effects or side effects coupled with social pressure, particularly husband/partner pressure to remove, would motivate women to seek early removal. Some women reported that their partners were concerned about the side effects that they were experiencing, and others described being forced to remove their implant by their partner.

This study also provides insight into why implant users seek removal before expiration.

Implant users’ experience and reporting of side effects appears to also influence their removal experience and explain why they do not always obtain removal on first attempt. Several women described being encouraged by their provider to keep their implant with counseling and side effect treatment. This provider reassurance is important for allaying health concerns associated with implant use and managing unwanted side effects. However, some women made subsequent attempts at removal. Our qualitative results suggest that removal to become pregnant or because a partner or husband demands removal appears to more likely to result in removal than complaints of side effects. Several providers said they would perform removal on request and that they believed that removal should be at the sole discretion of the woman using the implant. Some other providers and users noted that removal often also depended on provider judgment.

Although most women who had obtained an implant removal described their experience as easy, our qualitative findings indicate that difficult removals, including nonpalpable and bent/broken rods, occurred with some frequency. We cannot estimate prevalence of difficult removals from these data, but several women and most providers said that they had experienced a difficult removal. More research should explore the extent, causes, and consequences of difficult removals in this setting and elsewhere.

Finally, our data indicate that the financial cost of implant removal is uneven and can, in some cases, constrain access. Women seeking removal reported paying a range of fees despite the fact that GHS has a documented fee for removal of 2 Ghana cedis (approximately 40 US cents) and MSIG outreach has no fee. Both women and providers described that fees may differ based on the facility, the length of time a woman has had her implant, or the reasons for the removal. Such uneven charges for removal present a clear challenge to free and informed choice for contraceptive use.

Data indicate that the financial cost of implant removal is uneven and can sometimes constrain access.

GHS is committed to improving access to quality FP services to all Ghanaians and has prioritized the utilization of these study results to improve implant service provision. USAID Ghana is working with GHS to identify and implement interventions to improve the quality of implant service delivery and to ensure women have access to removal services for any reason and at no cost. Research findings have been disseminated throughout Ghana in a series of events culminating in a national steering committee meeting to synthesize recommendations. A technical working group convened to revise and update the national implant training materials, and a select group of national and regional FP trainers have now been trained on the updated materials.

### Limitations

Our study has several limitations. First, although the rsLog and client information center electronic databases provided a unique opportunity to identify and contact a large sample of implant users, the systems are both relatively new and the data they contained were not complete. In addition to incomplete or incorrect phone information, inherent challenges with a phone survey included phones being turned off, running out of battery, poor reception, numbers changing frequently, or respondents not answering calls.

We were also limited by how long the electronic systems had been in place. Most women recruited from the systems had had their implant for less than 18 months, so their experiences may be different from women who have had their implant for a longer amount of time. While the exit interviews provide perspectives from a few longer-term users, the limited sample makes drawing comparisons across groups difficult.

Finally, the eligibility criterion that women have phone information available in their record carries some risk of selection bias and likely may have excluded the poorest women. The experiences of some women who may have experienced challenges accessing removal services may have been missed or underrepresented.

## CONCLUSIONS

This study provides some reassuring data that most women who desire to remove their implant are able to receive a removal in a timely fashion. However, the results also indicate that not all women are able to access removal when and where they want and room for program improvement exists both in the public sector and for mobile outreach services.

## Notes

**Peer Reviewed**
